# UV-B Induced Generation of Reactive Oxygen Species Promotes Formation of BFA-Induced Compartments in Cells of *Arabidopsis* Root Apices

**DOI:** 10.3389/fpls.2015.01162

**Published:** 2016-01-13

**Authors:** Ken Yokawa, Tomoko Kagenishi, František Baluška

**Affiliations:** ^1^Institute of Cellular and Molecular Botany, University of Bonn, BonnGermany; ^2^Department of Biological Sciences, Tokyo Metropolitan UniversityTokyo, Japan

**Keywords:** UV-B, reactive oxygen species, root, negative phototropism, light-escape tropism

## Abstract

UV-B radiation is an important part of the electromagnetic spectrum emitted by the sun. For much of the period of biological evolution organisms have been exposed to UV radiation, and have developed diverse mechanisms to cope with this potential stress factor. Roots are usually shielded from exposure to UV by the surrounding soil, but may nevertheless be exposed to high energy radiation on the soil surface. Due to their high sensitivity to UV-B radiation, plant roots need to respond rapidly in order to minimize exposure on the surface. In addition to root gravitropism, effective light perception by roots has recently been discovered to be essential for triggering negative root phototropism in *Arabidopsis*. However, it is not fully understood how UV-B affects root growth and phototropism. Here, we report that UV-B induces rapid generation of reactive oxygen species which in turn promotes the formation of BFA-induced compartments in the *Arabidopsis* root apex. During unilateral UV-B irradiation of roots changes in auxin concentration on the illuminated side have been recorded. In conclusion, UV-B-induced and ROS-mediated stimulation of vesicle recycling promotes root growth and induces negative phototropism.

## Introduction

Due to their sessile nature, plants must accommodate changes in the light environment. Light is an essential physical factor in whole plant life cycle for photosynthesis and the regulation of plant development such as seed germination, cell elongation, organ tropisms, and flowering. Many proteins, including photoreceptors and other signaling molecules, are involved in the regulation of many physiological events as well as phenotypic plasticity in response to the light environment. Sunlight penetrating the Earth’s atmosphere contains a continuous spectrum of far- red and visible light as well as UV (ultra-violet) radiation. It has been reported that UV radiation affects plant development through various physiological processes (Frohnmeyer and Staiger, 2003; Jenkins, 2009). However, it is also well known that an excess amount of UV, containing high energy photons, damages the plant cells (Jansen et al., 1998). Free radicals, especially reactive oxygen species (ROS), are a typical by-product of the photo-excitation of certain compounds (Hideg and Vass, 1996; Allan and Fluhr, 1997; Pristov et al., 2013). ROS directly oxidize many biomolecules such as phospholipids in the plasma membrane, proteins, and nucleic acids, leading to severe damage to plant cells (Frohnmeyer and Staiger, 2003). However, in plant cells ROS also play an important role as signaling molecules in the effective regulation of cellular redox-homeostasis (Green and Fluhr, 1995; Hideg et al., 2013), as well as in promoting the biosynthesis of flavonoids which function as a sunscreen (Li et al., 1993; Hectors et al., 2012). In other words, UV-B induced ROS production seems to be involved in plant adaptation to UV radiation.

Since some decades ago, it has been revealed that roots are in fact light sensitive plant organs equipped with a range of photo-receptors and related signaling pathways (Feldman and Briggs, 1987; Kutschera and Briggs, 2012). Only one photoreceptor was known in 1971 whereas fourteen are discovered to date (Briggs and Lin, 2012). The root system possesses the same photoreceptors as the above-ground parts of the plant, which most likely allows roots to respond to light direction, intensity and wavelength (Chen et al., 2004; Briggs and Lin, 2012). It is well known that root-expressed phytochromes are involved in root hair formation, root growth and root gravitropism (De Simone et al., 2000; Correll and Kiss, 2005; Mo et al., 2015). Intriguingly, *Arabidopsis* roots also express the UV-B photoreceptor UVR8 (Rizzini et al., 2011) and a root-specific regulator, ROOT UVB SENSITIVE (RUS1 and 2) (Tong et al., 2008; Leasure et al., 2009); indicating that roots, like shoots, might possess a physiological mechanism for responding to external UV-B radiation.

In 1879, Francis Darwin was the first to discover negative phototropism of plant roots. One year later together with his father, Charles, Francis Darwin published the book, “The Power of Movements in Plants,” in which they not only describe both root and shoot tropisms, but also propose that some form of long-distance signaling can link the sensory organ apices with underlying basal tissues (Darwin, 1879, 1880). Since then research in plant physiology has resulted in the discovery of the signaling molecule, auxin, and provided us with insights into plant photo-reception. Specific photoreceptors enable plants to perceive and directionally respond to incoming light and this response is called phototropism. Growth toward a light source, as can be observed in shoots, is called positive phototropism, while bending away from the light source, as can be seen in roots, is called negative phototropism. We have previously reported that brief but strong blue light illumination (2 mW/cm^2^) of *Arabidopsis* roots induces immediate generation of ROS in root tips, resulting in an increase in root growth rate (Yokawa et al., 2011, 2013). This light-induced and ROS-enhanced root growth response is called escape tropism (Xu et al., 2013; Yokawa et al., 2013; for maize roots see Burbach et al., 2012) which, together with negative phototropism (Wan et al., 2012), enables *Arabidopsis* roots to effectively respond by avoiding unfavorable light conditions. It was also reported that PIN2 proteins (PIN-FORMED 2; auxin eﬄux carrier) expressed in root cells react to the light environment (Laxmi et al., 2008; Kagenishi et al., 2015). Wan et al. (2012) recently demonstrated that phototropin distribution in root cells is altered in response to blue light illumination (Wan et al., 2012). Interestingly, Dyachok et al. (2011) report that when COP1 (CONSTITUTIVE PHOTOMORPHOGENIC1) is activated by light, it dramatically enhances actin polymerization and *F*-actin bundling through regulation of the downstream ARP2/3-SCAR pathway in root cells, resulting in enhanced root elongation during the illumination period (Dyachok et al., 2011).

In nature, roots grow underground in the dark soil, to anchor the plant and absorb nutrients and water. Besides positive gravitropism, negative phototropism is essential to maintain appropriate root growth. Phototropism, like other root tropisms, requires polar auxin transport based on a high rate of endocytic vesicle recycling which relocates various membrane proteins including PINs (Blilou et al., 2005; Baluška et al., 2010; Wan et al., 2012; Baluška and Mancuso, 2013). Polar auxin transport, especially PIN2-based auxin transport toward the shoot, is sensitive to blue light which in turn is essential for differential cell growth controlling the underlying negative phototropism of *Arabidopsis* roots (Wan et al., 2012).

However, the mechanism that guides negative phototropism in response to UV-B irradiation is not yet understood. Here, we report that UV-B illumination induces almost immediate ROS generation at the root apex, resulting in the alteration of endocytic vesicle recycling. We have detected a change in the distribution of auxin in root apical cells during UV-B irradiation.

## Materials and Methods

### Plant Material

*Arabidopsis* (*Arabidopsis thaliana* L.) seeds were soaked in a sterilizing solution containing 4% sodium hypochlorite and 0.1% Triton X-100 for 15 min and washed at least five times with sterile distilled water. Sterilized seeds were planted on a 0.4% phytagel-fixed half-strength Murashige-Skoog growth medium without vitamin B. Petri dishes were incubated vertically at 23°C in darkness, in order to prevent the roots from acclimating to light.

### UV-B Treatment

For the UV-B treatments, all samples were irradiated with a UV-B 311 nm narrow band lamp (Philips, PL-S 9W/01/2P, Poland). The experimental UV-B intensity was measured and calibrated in each experiment with a UV-B broadband meter (Solarmeter model 6.0, Solartech, MI, USA). The values of UV-B described in the study are un-weighed UV-B intensity. For the UV-B treatments, the seedlings were placed between a microscope slide and a coverslip and kept in a vertical position. All root samples were irradiated from 20 to 30 cm distance of the light source (no heat transmission) with the cover slip in place, entirely blocking potential UV-C radiation emitted by the UV light source. This was confirmed with measurements using a spectrophotometer. For white light treatment, an array of light emitting diode (LED) was used to illuminate, and the irradiance was measured with photometer (HD2302.0, Delta Ohm) with a detector (LP471RAD, Delta Ohm). In the control treatment without UV-B radiation, the same UV-B lamp was covered with a polyester film to block UV-B radiation enabling the effect of any visible plus UV-A background illumination emitted by the lamp to be measured.

### Histochemical ROS Detection

Reactive oxygen species detection was carried out using histochemical staining methods. 3′, 3′-Diaminobenzidine (DAB) and Nitroblue tetrazolium (NBT) were used for detecting hydrogen peroxide and superoxide, respectively. For NBT staining, the seedlings were incubated in a solution of 1/10 MS medium for 10 min and then transferred to a 50 μM NBT solution dissolved in 1/10 MS medium for another 5 min at room temperature. Afterwards the samples were rinsed several times and the roots were irradiated with UV-B for 20 min. Likewise, DAB staining was carried out using the following procedure: the seedlings were incubated in a Tris buffer (pH 5.0) for 10 min and then infiltrated with the DAB solution (final concentration 0.7 mg/ml), for 5 min in a vacuum chamber at room temperature. The samples were washed twice with the Tris buffer. The roots were then treated with UV-B radiation for 20 min. The images of roots were captured with ×10 objective lens of a light microscope Leica DM750 (Solms, Germany). The staining intensity in root apex region was digitized and compared using densitometric method by ImageJ software (ver. 1.43u for Macintosh OSX).

### Confocal Microscopy for Monitoring Endocytic Vesicle Recycling Activity

To monitor the vesicle recycling in root cells, roots were treated with Brefeldin-A (BFA), which binds ARFGNOM; an inhibitor of exocytosis, and FM4-64 fluorescence dye to visualize the plasma membrane. A BFA stock solution was prepared in DMSO at 35 mM concentration. Since the root apical region is very delicate and sensitive to stress, the procedure for monitoring endocytic vesicle recycling was executed very carefully. Firstly, seedlings were placed between a microscope slide and a cover slip and the gap in between was then filled with 1/10 MS medium and kept in a vertical position. The seedlings were incubated for at least 1 h in darkness to reduce any endocytic activity in the root cells. After incubation, the MS medium was removed carefully with small strips of filter paper and replaced by the FM4-64 solution. The roots were then incubated for 10 min to allow proper staining. The FM4-64 solution was then replaced with the BFA solution (final concentration 35 μM) as described above. The roots were then irradiated with UV-B (UV-C region was completely filtered by the glass cover slip) for 20 min. After the treatment with BFA and UV-B, the seedlings were rinsed with 1/10 MS medium and observed under a confocal laser-scanning microscope (FV-1000, Olympus). Images of FM4-64 fluorescence were captured using excitation and emission wavelengths of 514 and 640-nm, respectively, under ×40 magnification with an oil-immersed lens.

Auxin distribution altered by unilateral UV-B illumination was visualized using the transgenic *Arabidopsis* line DII-VENUS with a laser-scanning confocal microscope (Brunoud et al., 2012). The concentration of auxin in each cell is reflected by the fluorescence of the nuclei. The samples of the DII-VENUS line were stained with FM4-64 to visualize cell membranes. The excitation and emission wavelengths used to analyze fluorescence in the DII-VENUS line were 515 and 528-nm, respectively (FM4-64 staining carried out as above). Data quantification of the microscope images was performed using ImageJ (ver. 1.43u for Macintosh OSX).

### Image Analysis of BFA-Induced Compartments and DII-VENUS Fluorescence

On computer display, the diameter of BFA-induced compartments visualized with FM4-64 was measured using ImageJ software manually. BFA-induced compartments found in the epidermal cells in transition zone, between meristem and elongation zone, was measured and averaged. Small vesicles (less than 1 μm diameter) were not counted.

For the comparison of DII-VENUS fluorescence, the fluorescence images of green channel (VENUS) taken under the confocal were converted into inverted gray scale using ImageJ. Either left or right side of epidermal cells in root transition zone was selected and total pixels were counted. The ratio of fluorescence intensity between two sides of roots were calculated and averaged to quantify the changes of auxin distributions.

### Statistic Analysis

All numerical data obtained here were analyzed and tested in appropriate statistical methods. Student’s *t*-test was applied to test a level of significance using Microsoft Excel 2011, and for the comparison of the effects of either UV-B on BFA-compartment formation in **Figure 3** or three different diphenyliodonium (DPI) concentrations in **Figure 4**, Tukey’s HSD (honestly significant difference) was applied to test a level of significance at *p* < 0.05 using R software (R for Mac OS × Cocoa, http://www.R-project.org).

## Results

### *Arabidopsis* Root Growth is Promoted by UV-B Treatment

**FIGURE 1:**
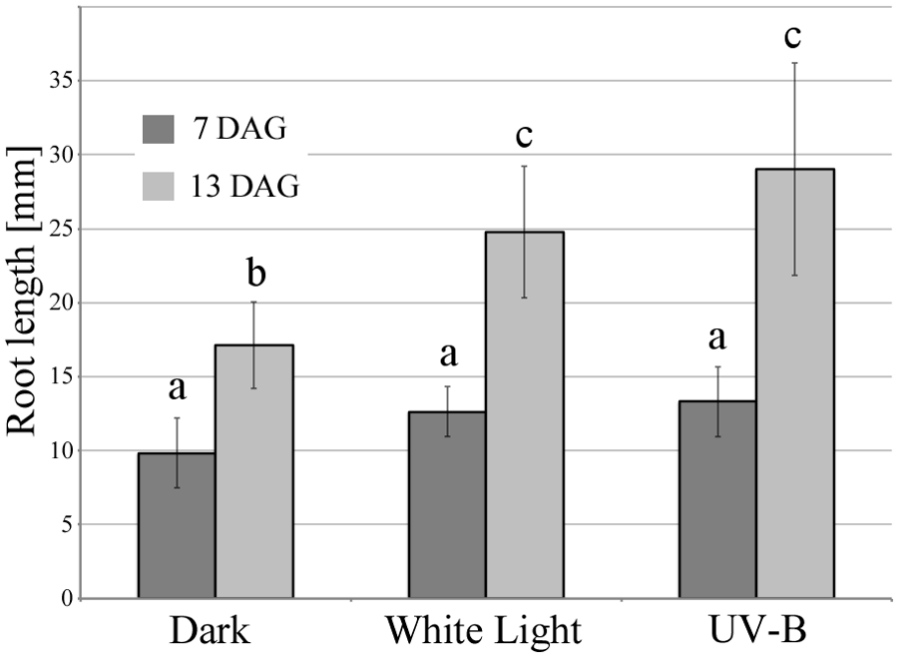
**UV-B and white light-induced primary root elongation.** Primary root length was measured at 7 and 13 days after germination. The light and dark period was 16 and 8 h, respectively. White light irradiance was 2 mW/cm^2^ (*c.* 19.2 kJ/m^2^/day) and UV-B was 0.3 mW/cm^2^ (*c.* 6.7 kJ/m^2^/day). Error bars indicate ±1 standard error. (*n* = 14) Different letters on the bars indicate significant difference tested by one-way ANOVA, *p* < 0.05.

As described above, the illumination of roots stimulates primary root growth. **Figure 1** shows that the growth rate of *Arabidopsis* roots was enhanced by periodic treatment with white light as well as under UV-B irradiation. Starting from 7 days after germination, root growth rate was increased compared to control roots grown in darkness. To assess the effect of low-dose of lights on root growth, periodical irradiation was conducted. UV-B and white light was programmed to irradiate samples incubated in dark chamber at room temperature for 1 min at the beginning of every hour 16 times in a day (8 h were dark cycle). The intensities of UV-B and white light were at 0.3 mW/cm^2^ (*c.* 6.7 kJ/m^2^/day) and 2 mW/cm^2^ (*c.* 19.2 kJ/m^2^/day). As indicated previously, *Arabidopsis* roots display escape tropism by bending away from the UV-B source due to an enhanced root growth rate. The UV-B irradiance dose used here is equivalent to that occurring naturally, and it stimulates negative root phototropic responses, whereas root growth is strongly inhibited under high UV-B irradiance.

### UV-B Irradiation Triggers Rapid ROS Production in Root Apices

**FIGURE 2:**
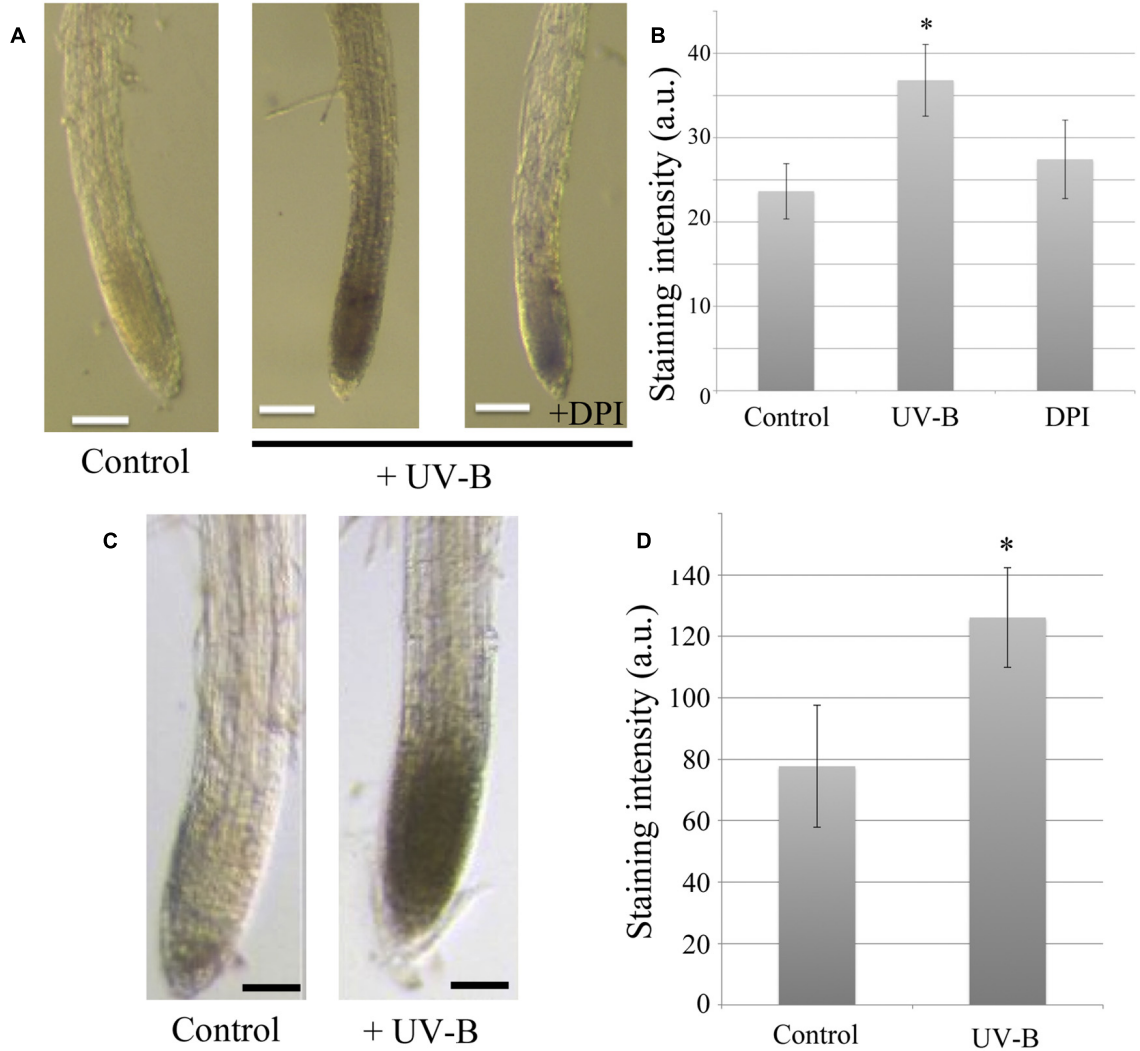
**UV-B induced reactive oxygen species (ROS) generation in the root apex. (A)** Representative photographs of superoxide generation at the root apex detected by NBT staining and treatment of 10 μM DPI (flavo-protein inhibitor). UV-B dose was 0.3 mW/cm^2^ for 20 min. Scale bars indicate 20 μm. **(B)** Comparison of NBT staining intensity. Error bars indicate ±1 standard error. (*n* = 5; **C**) Representative photographs of hydrogen peroxide production at the root apex detected using DAB staining. UV-B dose was 0.3 mW/cm^2^ for 20 min. Bars indicate 20 μm. **(D)** Comparison of DAB staining intensity. Error bars indicate ±1 standard error. (*n* = 12) Asterisk on the bars indicates significant difference tested by *t*-test, *p* < 0.05.

To identify whether ROS function as primary signaling molecules in the UV-B-provoked root response, NBT and DAB histochemical staining methods were applied. NBT and DAB reagents react with superoxide and hydrogen peroxide, respectively. These chemicals immediately form a colored precipitate upon contact with the free radicals, which can then be visualized by microscopy. Strong NBT staining patterns were present in the region of the root apex after 20 min of UV-B irradiation at 0.3 mW/cm^2^ (**Figure 2**). This illustrates that irradiation with UV-B induced the generation of superoxide in the root apex, which is metabolically very active compared to other parts of the root. In addition, staining intensities reflecting ROS concentration are reduced by treatment with DPI, which is commonly used as an inhibitor of flavo-proteins, including NADPH oxidase (**Figure 2A**). Although DPI inhibits the function of membrane-associated NADPH oxidase, ROS formation was nevertheless found in *rhd2-4* mutants (NADPH oxidase-deficient mutant, data not shown). Hence, the source of ROS generation must be another light-absorbing molecule containing a flavin group, because DPI also inhibits flavin-mediated electron transfer. Allan and Fluhr (1997) suggested that UV-B-induced ROS generation is likely to originate from specific flavo-proteins. Staining with DAB enabled us to detect hydrogen peroxide production which occurred in the same root region as superoxide production (**Figure 2B**). This suggests that the superoxide generated by UV-B is immediately converted into hydrogen peroxide by superoxide dismutase (SOD).

### UV-B Activates Endocytic Vesicle Recycling in Cells of *Arabidopsis* Root Apices

**FIGURE 3:**
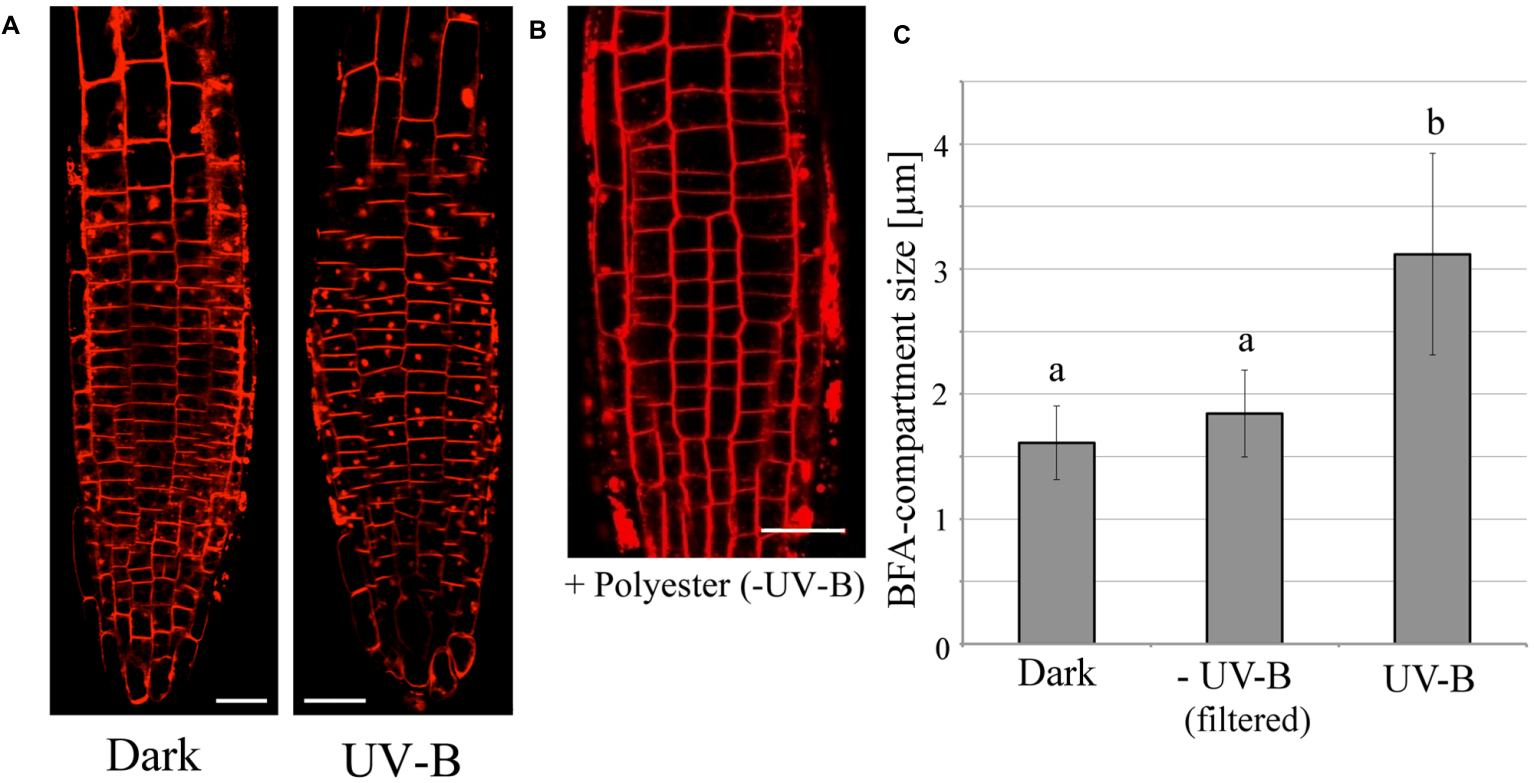
**The promotion of endocytic vesicle recycling during treatment with UV-B irradiance (0.3 mW/cm^2^) for 20 min and Brefeldin-A (BFA). (A)** Representative fluorescence images of UV-B-induced formation of BFA-compartments in root tip epidermal cells. Red spots represent BFA-compartments. Plasma membranes were stained with FM4-64 fluorescent dye. BFA concentration was 35 μM. **(B)** The effect of background illumination emitted by the UV-B lamp covered with a polyester film. Scale bars indicate 20 μm. **(C)** Comparison of the size of BFA-induced compartments in root epidermal cells. Roots were irradiated for 20 min with UV-B with or without polyester film. BFA compartments (33 in control, 104 in UV-B, 69 in UV-B with cutoff filter from 3 to 10 independent treated roots) were counted and their diameters were averaged. Different letters on the bars indicate significant difference tested by Tukey’s HSD test, *p* < 0.05.

We assessed the effect of UV-B radiation on endocytic vesicle recycling in root cells, because vesicle recycling plays a vital role in polar auxin transport involved in root tropisms. The rate of endocytic vesicle recycling can be visualized using the Brefeldin-A (BFA) reagent, which inhibits exocytosis and leads to the formation of a spherical structure in the cytosolic space called BFA-compartment. After 20 min of UV-B treatment (0.3 mW/cm^2^), BFA-compartments formed in root epidermal cells of the transition zone (**Figure 3A**), whereas they were not formed in the control plants kept in darkness. Importantly, the endocytic formation of the BFA-induced compartments in response to UV-B did not occur in the UV-B control treatments, when the UV-B lamp was covered with a polyester film. This shows that the background illumination (ranging from UV-A to all visible wavelengths) emitted by the lamp did not alter root cellular responses (**Figure 3B**). These results indicate that the increase in the rate of vesicle recycling under UV-B radiation is an integral part of negative root tropism to avoid UV-B radiation.

### ROS Enhances Endocytic Vesicle Recycling in Cells of *Arabidopsis* Root Apices

**FIGURE 4:**
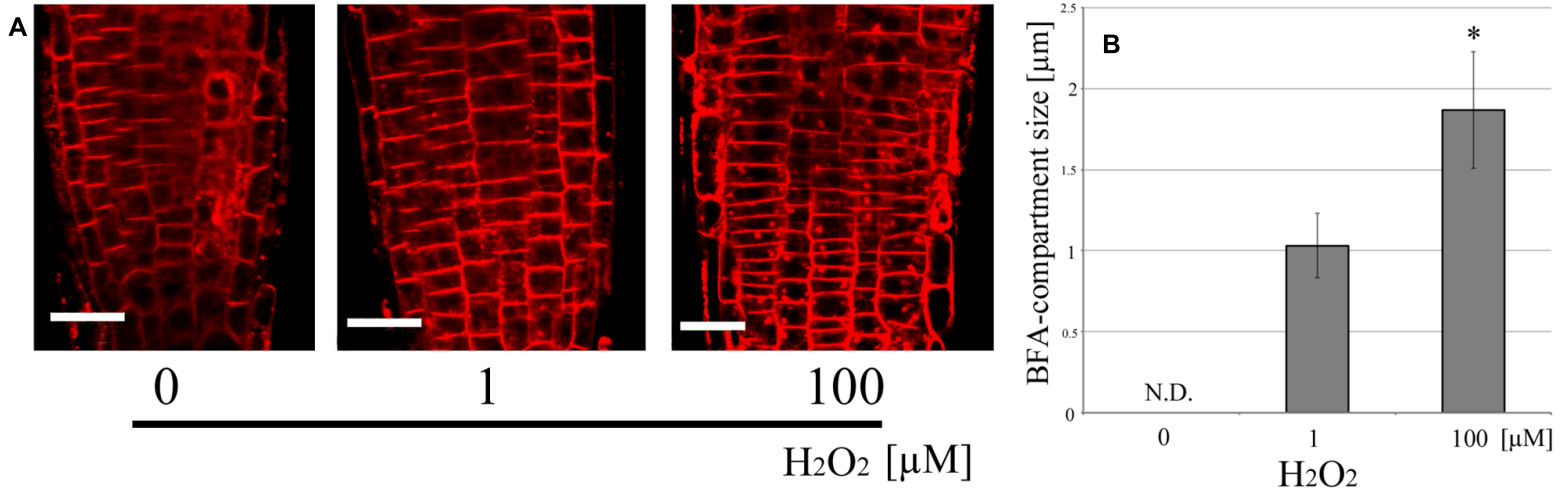
**Endocytic vesicle recycling induced by exogenously applied H_2_O_2_. (A)** Representative fluorescence images of the effect of H_2_O_2_ on endocytic vesicle recycling. **(B)** Comparison of the size of BFA-compartments in root epidermal cells. Roots were incubated for 20 min. 100 μM of H_2_O_2_ promoted vesicle recycling. BFA compartments (50 in 1 μM, 46 in 100 μM from five independent treated roots) were counted and their diameters were averaged. Scale bars indicate 20 μm. Asterisk on the bars indicates significant difference tested by *t*-test, *p* < 0.05.

**FIGURE 5:**
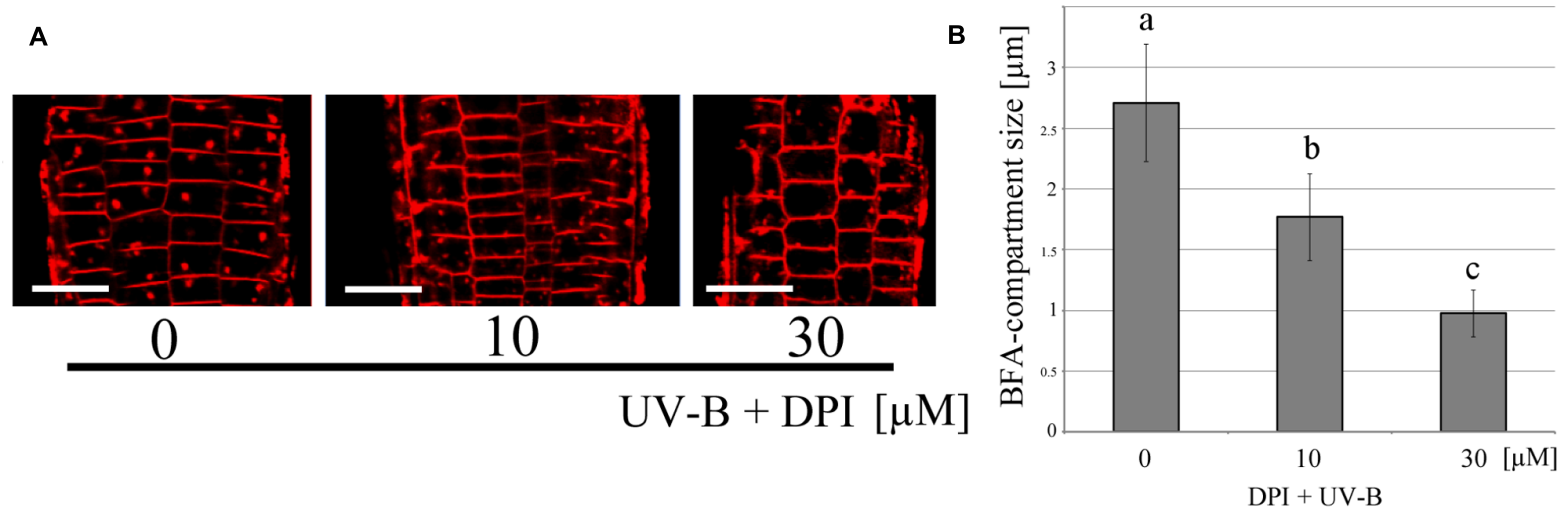
**Inhibition of UV-B-induced endocytic vesicle recycling by DPI. (A)** Representative fluorescence images of endocytic vesicle recycling following DPI application. Scale bars indicate 20 μm. **(B)** Comparison of BFA-compartment size in root epidermal cells. Roots were irradiated with UV-B for 20 min in the presence of DPI. 30 μM of DPI inhibited vesicle recycling. BFA compartments (99 in 0 μM, 60 in 10 μM and 66 in 30 μM from eight independent treated roots) were counted and their diameters were averaged. Error bars indicate standard error. Different letters on the bars indicate significant difference tested by Tukey’s HSD test, *p* < 0.05.

In order to clarify the relationship between ROS and vesicle recycling, the BFA-induced compartments in roots were monitored after the treatment of exogenously applied hydrogen peroxide (H_2_O_2_) which induces oxidative stress (**Figure 4**). The number of BFA compartments was increased by the treatment of H_2_O_2_ at a concentration of 100 μM in the absence of UV-B, indicating that ROS might affect polar auxin transport and root tropism because endocytic vesicle recycling plays important roles in these processes. Additionally, treatment with the flavo-protein inhibitor DPI, illustrated the effect of UV-B radiation on the formation of BFA-induced compartments. The size of these BFA compartments was significantly decreased in root cells treated with 30 μM DPI and UV-B (20 min, 0.3 mW/cm^2^) (**Figure 5**). Based on the results from the DPI treatment, there was an NADPH oxidase- or other flavoprotein-dependency of the UV-B-induced generation of ROS. Alternatively, other physiological mechanisms affected by DPI might alter the formation of BFA-induced compartments. Taken together, UV-B induces ROS production, and ROS promote the formation of BFA-induced compartments in cells of root apices.

### Unilateral UV-B Irradiation Alters the Distribution of Auxin in *Arabidopsis* Root Apex Cells

**FIGURE 6:**
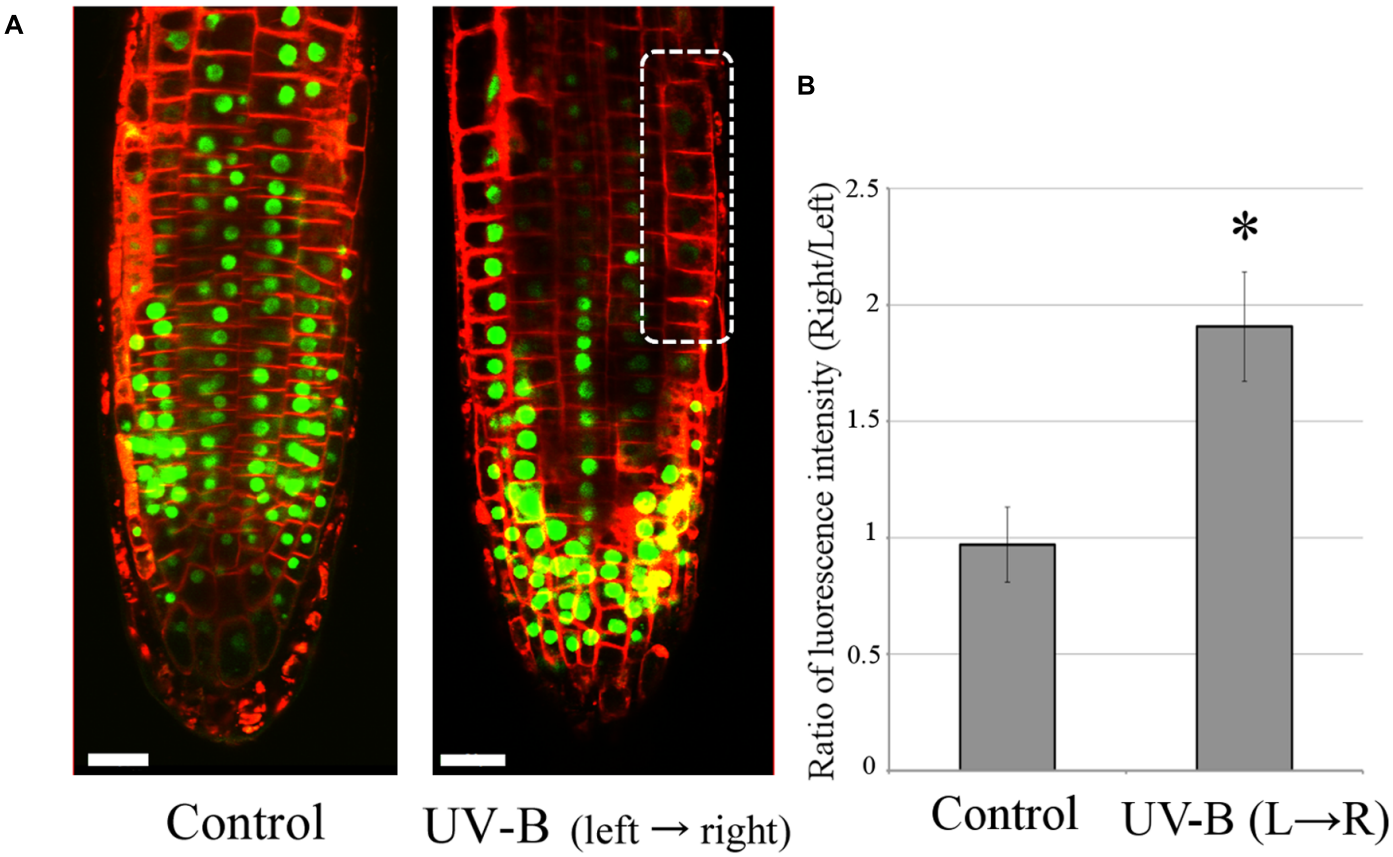
**Unilateral UV-B irradiation (0.3 mW/cm^2^ for 20 min) alters auxin distribution in root tips.** The direction of UV-B radiation is from left to right. **(A)** Auxin concentrations were visualized using the transgenic *Arabidopsis* line DII-VENUS. Decrease of VENUS fluorescence (green signal) indicates an elevation of cellular auxin concentration. The dotted circle marks the region with the highest auxin concentration (*n* = 3). The representative fluorescence images are shown here. Plasma membranes were stained with FM4-64 fluorescent dye in red color. Scale bars indicate 20 μm. **(B)** The comparison of ratio of VENUS fluorescence between left and right side of root epidermal cells in transition zone. Error bars indicate ±1 standard error. (*n* = 3) Asterisk on the bars indicates significant difference tested by *t*-test, *p* < 0.05.

The transgenic *Arabidopsis* line DII-VENUS (Brunoud et al., 2012) was used to analyze the auxin distribution in root apices. It is a useful tool for visualizing the changes in auxin concentration in single root cells under the laser-scanning confocal microscope. Fluorescence intensity of nuclei in DII-VENUS root cells decreases with rising auxin concentrations. **Figure 6** depicts auxin re-distribution to the shaded side of the root after 20 min of unilateral UV-B irradiation at 0.3 mW/cm^2^ (light source on the left hand side). Since a high concentration of auxin inhibits root growth, this nicely demonstrates negative phototropism, that is root bending away from the light source. In addition, *Arabidopsis* roots of both the Columbia wild-type and several mutant lines, *uvr8-6* (UVR8 mutant), *phot1/phot2* (PHOT1/2 mutant) and *cop1-4* (COP1 mutant), have produced the same root growth pattern of UV-B avoidance when unilaterally irradiated for 48 h at 0.01 mW/cm^2^ (data not shown). The results presented here indicate that *Arabidopsis* roots have the ability to detect the direction of incoming UV-B radiation and avoid it, reacting by altering the polar flow of auxin in the root apical region.

## Discussion

### *Arabidopsis* Roots are Extremely Sensitive to Light, Especially to the UV-B Wavelengths

Why should plant roots be so sensitive to light? In nature, root systems are typically in the soil (underground and in darkness) anchoring the plant in the ground, as well as absorbing nutrients and water. Roots possess complex signaling pathways and specific receptors fine-tuned to sense changes in their environment (Malamy, 2005; Forde and Walch-Liu, 2009; Monshausen and Gilroy, 2009; Baluška and Mancuso, 2013). It is well known that germinating roots grow into the soil using positive gravitropism. In addition to the perception of gravity, it has recently been shown that roots are also capable of very sensitive photoperception. This is illustrated by the fact that even a few seconds of exposure to light result in a burst of ROS production (Yokawa et al., 2011, 2013). Moreover, root cells have a similar set of photoreceptors to the above-ground parts of the plant (Briggs and Lin, 2012), and these enable them to avoid light.

Besides during seed germination, when the emerging roots are often exposed to light, strong wind or earthquakes may unearth roots exposing them to sunlight. Hence, it is not surprising that roots are sensitive to light which stimulates a rapid response directed at returning the root to the soil. High energy photons in the UV region of the solar spectrum make it the most damaging to biomolecules, especially DNA, whose nucleotides have an absorption maximum close to the boundary of the UV-B and UV-C wavebands (Björn, 2008). This would explain why plants have developed effective mechanisms for protecting themselves from UV radiation. Furthermore, *Arabidopsis*, and presumably other plants too, possess a root-specific UV-B signaling pathway controlled by RUS1 and RUS2 proteins (Tong et al., 2008; Leasure et al., 2009), strongly suggesting that roots have evolved in an environment subjected to UV-B radiation and are able to respond to and avoid UV-B radiation. Interestingly, both RUS1 (Yu et al., 2013) and RUS2 (Ge et al., 2010) are essential for polar auxin transport in root apices of *Arabidopsis*.

### Light-Induced ROS and Auxin Drive Escape and Avoidance Tropisms of *Arabidopsis* Roots

Laxmi et al. (2008) reported that roots of *Arabidopsis* grow faster under illumination which is accompanied by higher PIN expression levels and increased rates of endocytic vesicle recycling. PIN2, which is critical to almost all root tropisms, is especially sensitive to light (Laxmi et al., 2008; Sassi et al., 2012; Wan et al., 2012; Mo et al., 2015). It was also demonstrated that root-localized phytochrome and their precursor PΦB impact on enhanced root elongation under white, blue, and red light conditions (Costigan et al., 2011; Warnasooriya and Montgomery, 2011). We have previously reported that illumination of roots induces an immediate burst of ROS in root apex cells of *Arabidopsis* (Yokawa et al., 2011, 2013). As the experiments shown in **Figure 2**, root cells irradiated with UV-B induced ROS production especially in apex region, and this pattern is very similar in blue-light illuminated roots (Yokawa et al., 2011). Surprisingly, ROS are also crucial signaling molecules in adaptive stress responses (Green and Fluhr, 1995; Rao et al., 1996; Egert and Tevini, 2003; Hideg et al., 2013; Wrzaczek et al., 2013). In general, ROS are known to induce expression of many genes involved in stress responses allowing plants to cope with a challenging environment. In root apex, the transition zone is localized between the apical meristem and the zone of elongation (Verbelen et al., 2006) and plays an important role in many tropisms which in turn are driven by a high intercellular flux of auxin (Baluška et al., 2005, 2010; Baluška and Mancuso, 2013). Furthermore, it has been reported that ROS are also generated in response to several other stimuli such as gravity and touch (Joo et al., 2001; Chehab et al., 2009), especially in cells of the transition zone. In this study, we showed the acceleration of BFA-induced compartment formation by either UV-B irradiation (**Figure 3**) or only applying exogenous H_2_O_2_ as a ROS source (**Figure 4**). It indicates that robust UV-B-induced ROS production was evident in cells of the transition zone, suggesting that this region is highly sensitive to external UV-B radiation. However, further studies an additional experiments, such as BFA washout, are required to monitor how UV-B changes entire endo/exocytic vesicle recycling apparatus in cells of the root apex transition zone.

Additionally, the transition zone is particularly sensitive to auxin, which displays particularly high flux rates (Mancuso et al., 2005; Wan et al., 2012). The ROS generated act downstream of auxin (Ivanchenko et al., 2013). Here we report that ROS formation is induced by UV-B radiation, similarly as by blue light, and stimulate endocytic vesicle recycling as well as auxin transport between irradiated and shaded side of the transition zone (**Figure 6**; for blue light see Wan et al., 2012). Importantly, this specific zone of the root apex determines the direction of root growth through the differential release of auxin vesicles into the region of cell elongation (Baluška et al., 1994, 1996, 2004, 2010).

What is a source of ROS controlling UV-B mediated endocytic vesicle recycling and auxin redistribution? As **Figures 2A,B** shows, the generation of superoxide and H_2_O_2_ were detected after 20 min of UV-B irradiation. As a possibility of enzyme-mediating reaction, NADPH oxidase on the plasma membrane would be a candidate as a ROS generator. It is well studied as an important enzyme that produces superoxide in response to many physiological events including abiotic- or biotic- stresses (Miller et al., 2009; Marino et al., 2012). Here we treated DPI, diphenylene iodonium; a NADPH oxidase inhibitor, with UV-B irradiated roots. It reduced superoxide accumulation (**Figure 2A**) and the rate of vesicle recycling (**Figure 5**) under UV-B treatment. However, since DPI functions to inhibit an electron transfer of flavoproteins (NADPH oxidase is one of them), we cannot rule out a possibility of other radical generating reaction via flavin-containing biomolecules (O’Donnell et al., 1994). UV-B itself has a high energy compared to visible wavelengths of light and is likely enough to excite many molecules. In blue light region, there are many reported studies indicating that flavin-containing molecules immediately generate radicals and ROS *in vivo* by absorbing light (Massey et al., 1969; Prolla and Mehlhorn, 1990; Hockberger et al., 1999). Intriguingly, it was reported that irradiating blue light to tryptophan solution *in vivo* produced a precursor of auxin, *indole-3-acetaldehyde*, and it might be relevant to rapid phototropism in plants (Koshiba et al., 1993; Yokawa et al., 2014a). We also hypothesized that internalized pectin via endocytosis can also be a factor controlling ROS homeostasis under UV-B environment (Yokawa and Baluška, 2015). ROS is ultimately unstable chemical species. However, it has an advantage to be generated immediately on site, and can trigger downstream signaling through chemical reactions. The mechanism of light-promoted ROS production must be elucidated.

### Roots Exhibit Avoidance to Light via Rapid and Slow Responses

**FIGURE 7:**
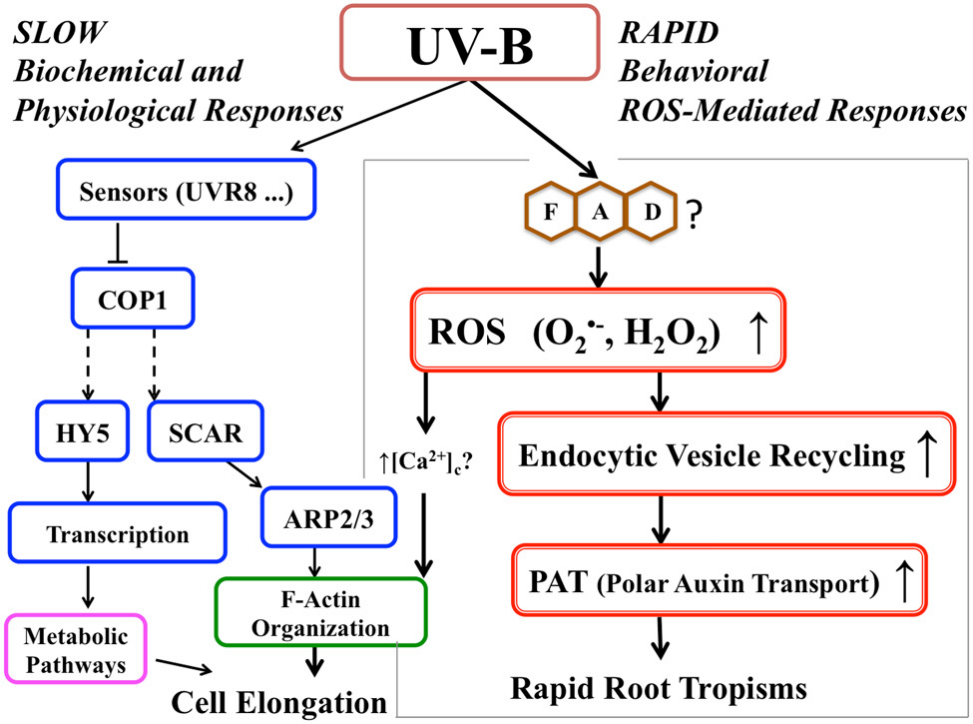
**Proposed signaling pathway for root avoidance responses to UV-B irradiance.** The left-hand side of the scheme depicts the UV-B-specific receptor-dependent signaling pathway (‘slow biochemical and physiological response’). The right-hand side shows ROS-driven speeding-up of the endocytic vesicle recycling and polar auxin transport (‘rapid response’) leading to the escape phototropism.

This is the first demonstration that ROS produced by UV-B exposure in the root apical cells are involved in general acceleration of endocytic vesicle recycling. Increased vesicle recycling leads to avoidance tropism in roots presumably caused by differentially increased polar auxin transport. This response can be considered a type of plant tropism that requires rapid responses. Plants cannot wait until proteins are ready to respond to stress situations. They are utilizing dual mode of actions, (1) quick behavior as shown in this report, and (2) slow changes of the physiological conditions which is mainly for preparing for upcoming events. Similar avoidance tropism was also reported for *Arabidopsis* roots exposed to salt stress (Li and Zhang, 2008; Sun et al., 2008; Galvan-Ampudia et al., 2013; Rosquete and Kleine-Vehn, 2013). Moreover, the illumination of roots changes their response to salt stress environment (Yokawa et al., 2014b). In the case of the UV-B escape tropism, this is based on UV-B-induced negative phototropism combined with general root growth acceleration (escape tropism). As summarized in **Figure 7**, this rapid response of roots to UV-B, mediated by ROS signaling molecules, allows efficient and very rapid root escape tropism. However, identifying sources of UV-B-induced ROS and the detailed mechanism of how the ROS signal directly stimulates vesicle recycling are important issues which will need to be elucidated in the future.

## Author Contributions

KY and TK conducted and analyzed the experiments.

KY, TK, and FB contributed to design of the experiments and composition of the manuscript.

## Conflict of Interest Statement

The authors declare that the research was conducted in the absence of any commercial or financial relationships that could be construed as a potential conflict of interest.
